# High-performance supercapacitors based on the carbon nanotubes, graphene and graphite nanoparticles electrodes

**DOI:** 10.1016/j.heliyon.2018.e00862

**Published:** 2018-11-20

**Authors:** L. Fekri Aval, M. Ghoranneviss, G. Behzadi Pour

**Affiliations:** a Plasma Physics Research Center, Science and Research Branch, Islamic Azad University, Tehran, Iran; b Department of Physics, East Tehran Branch, Islamic Azad University, Tehran, Iran

**Keywords:** Materials science, Electrical engineering, Energy

## Abstract

In this study, the three structures of the symmetric paper supercapacitors based on the carbon nanotubes (CNTs), graphite nanoparticles (GNPs) and graphene electrodes have been fabricated. In the supercapacitors was used of polyvinyl alcohol (PVA)/phosphoric acid (H_3_PO_4_) as a gel electrolyte and the BaTiO_3_ film as a separator film. The carbon nanomaterials, gel electrolyte surface, and electrode films were characterized by scanning electron microscopy (SEM) and transmission electron microscope (TEM). The specific capacitance of the symmetric paper supercapacitors using charge-discharge technique and C-V curves at the voltage scan rates 20 mV/s and 150 mV/s have been investigated. The symmetric paper supercapacitor based on the CNTs electrode showed higher specific capacitance 411 F g^−^^1^, compared to GNPs and graphene supercapacitors. Also by electrochemical impedance spectroscopy, the Nyquist curves of the symmetric paper supercapacitors have been plotted. For the symmetric paper supercapacitors based on the GNPs, graphene and CNTs electrodes the equivalent series resistance (ESR) resistance was 210 Ω, 96 Ω and 101 Ω respectively. The flexible symmetric paper supercapacitor based on BaTiO_3_/PVA/CNTs structure denotes a new type of the flexible supercapacitor that can be applied to the soft electronic.

## 1 Introduction

Supercapacitors with high power density and storage capacity are devices for electrical energy storage. A large application of supercapacitors is needed in the transport and hybrid electric vehicles. Supercapacitors are used in electronic devices such as power supply stabilizer, flashes deliver power, grid power buffer, energy harvesting and Energy recovery. The flexibility of paper supercapacitor allows the ability of rolling energy storage device or folding up to obtain high efficiency. Paper supercapacitors are commonly used in MEMS-based, transistors, sensors and solar cells systems. Based on the charge storage mechanism supercapacitors are divided into pseudocapacitors, electrical double layer capacitors and hybrid supercapacitors [1, 2, 3]. Recently, the electrodes of electrical double layer supercapacitors are fabricated of CNTs, GNPs, and graphene due to light-weight, electrochemical stability, and excellent conductivity. CNTs because of high electrical conductivity could replace collectors in supercapacitors. The larger specific surface area (SSA) of CNTs (430–1600 m^2^g^−^^1^) as an electrode in supercapacitor display the higher specific capacitance. The solution of polyvinyl alcohol (PVA) and phosphoric acid (H_3_PO_4_) are used as gel electrolyte in paper supercapacitors. In Ref. [4] the paper supercapacitor based on CNTs electrodes and H_3_PO_4_/PVA gel electrolyte has been described. That paper showed the specific capacitance of the supercapacitor was 47 Fg^−^^1^. A solid-state paper based CNTs supercapacitor with H_3_PO_4_/PVA electrolyte has been studied by Rajmani et al. [5]. They have shown the specific capacitance was 115 Fg^−^^1^. Graphene is a single layer with unique properties such as high surface-to-volume ratio and excellent conductivity and is suitable for energy storage due to their rate/cyclic capability and improved capacity [6]. The surface area of a single graphene sheet is 2630 m^2^/g [7]. Supercapacitors based on the graphene material are usually contained porous graphene, graphene foams, graphene fibers, graphene aerogels, graphene films, reduced graphene oxide, graphene/CNT, graphene/conducting polymers and graphene/oxides [8, 9, 10, 11, 12, 13, 14]. Kumar et al. [15] investigated the high-performance supercapacitor based on the Fe_3_O_4_/reduced graphene oxide nanosheets hybrid electrode. They showed the specific capacitances of 3D hybrid materials was 455 F g^−1^ at the scan rate of 8 mV s^−1^. In another study, self-assembled hierarchical formation of conjugated 3D cobalt oxide nanobead−CNT−graphene nanostructure for supercapacitor electrode has been reported in Ref. [16]. That paper indicated for the 3D cobalt oxide nanobead−CNT−graphene electrode the specific capacitance of the supercapacitor was 600 Fg^−^^1^ at the charge/discharge current density of 0.7Ag^−^^1^. Li et al. [17] showed the specific capacitance of the paper supercapacitor with CNTs/graphene composites was 100 Fg^−^^1^. A supercapacitor based on graphene foam/polyvinyl alcohol/formaldehyde (GF/PVA-F) has been presented in Ref. [18]. That paper described the maximum energy density and power density were 42 mWh cm^−2^ and 0.5 W cm^−^^2^ respectively. In this research, design, and fabrication of different paper supercapacitor based on CNTs, graphene and graphite nanoparticles electrodes and gel electrolyte have been investigated. The H_3_PO_4_/PVA was used as a gel electrolyte and BaTiO_3_ was used as a separator. The different films were characterized using the SEM analyze. We compared the effects of different electrodes on the electrical properties of the symmetric paper supercapacitors. Also, the specific capacitance of the symmetric paper supercapacitors using cyclic voltammetry and galvanostatic techniques were investigated.

## 2 Materials and methods

At first, on the paper substrate using the push coating method a thin layer of mixture BaTiO_3_ (233.2 g mol^−^^1^, Merck brand, 3 gr) and N-Methyl-2-pyrrolidone (99.13 g mol^−^^1^, Merck brand, NMP: 10 mL) has been coated on the substrate. The paper with separator was dried using the oven at 60 °C and for 15 min. PVA is a polymer with excellent mechanical properties that is used as both separator and electrolyte in supercapacitors [4]. The gel electrolyte films of paper supercapacitors were prepared by mixing PVA (278.34 g mol^−^^1^, Sigma Aldrich brand) and H_3_PO_4_ (98 g mol^−^^1^). At first, the PVA (1 g) and deionized water (10 mL) were mixed using magnetic stirring for 2 h at 90 °C. Then, the H_3_PO_4_ (4.7 g) was added into the mixture. Using the push coting method on the PVA film a layer of carbon nanomaterials has been coated. The CNTs was prepared by mixing sulfuric acid (98.078 g mol^−^^1^) and nitric acid (163.01 g mol^−^^1^) (3 H_2_SO_4_: 1 HNO_3_). This solution was dispersed by magnetic stirring for 2 h at 90 °C. The adhesion between the gel electrolyte film and the electrode (GNPs and graphene) prevents the carbon nanomaterials peeled off from the surface after bending the paper supercapacitor. The cyclic voltammetry and charge-discharge techniques and also impedance measurement of the symmetric paper supercapacitors have been done using potentiostat/galvanostat model ZIVE SP1. The frequency range of ZIVE SP1 is 10 μHz to 1 MHz with frequency accuracy 0.01%. The current range of ZIVE SP1 is 1 nA to 1 A.

## 3 Results and discussion

**Fig. 1:**
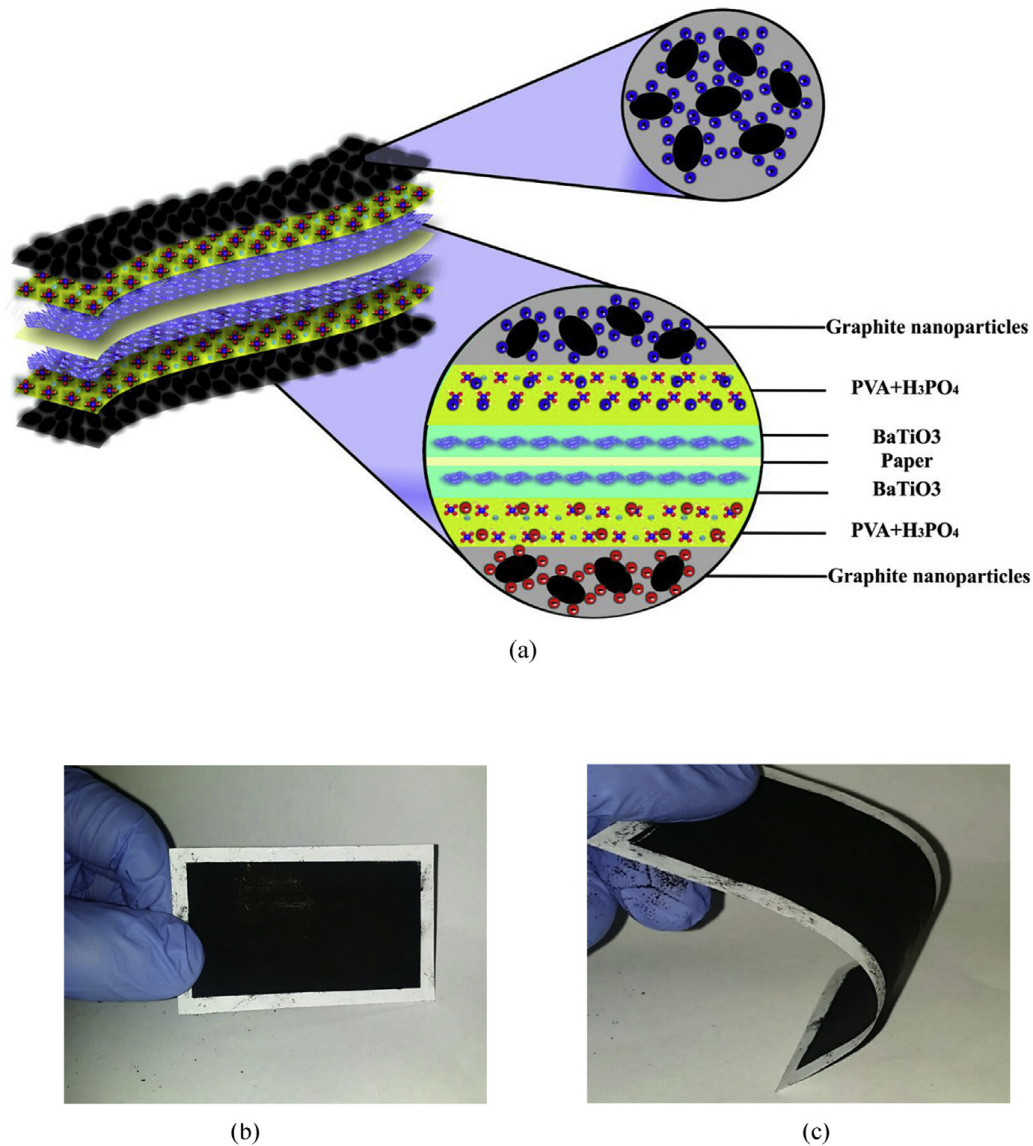
(a) schematic of the symmetric paper supercapacitor with the structure of GNPs electrodes (b) image of the fabricated supercapacitor (c) image of paper supercapacitor in bend state.

**Fig. 2:**
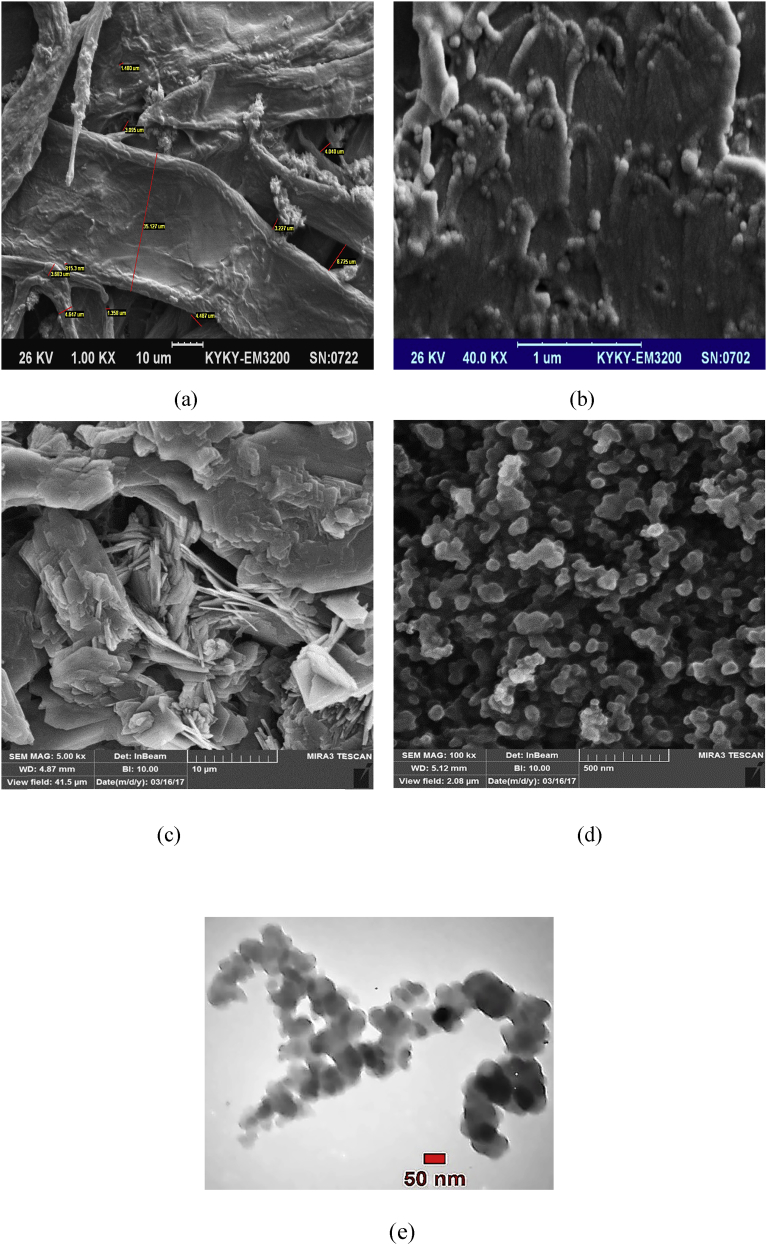
SEM images of paper surface (a) before and (b) after BaTiO_3_ gel separator coating (c) PVA gel electrolyte surface (d) GNPs electrode surface (e) TEM image of GNPs.

**Fig. 3:**
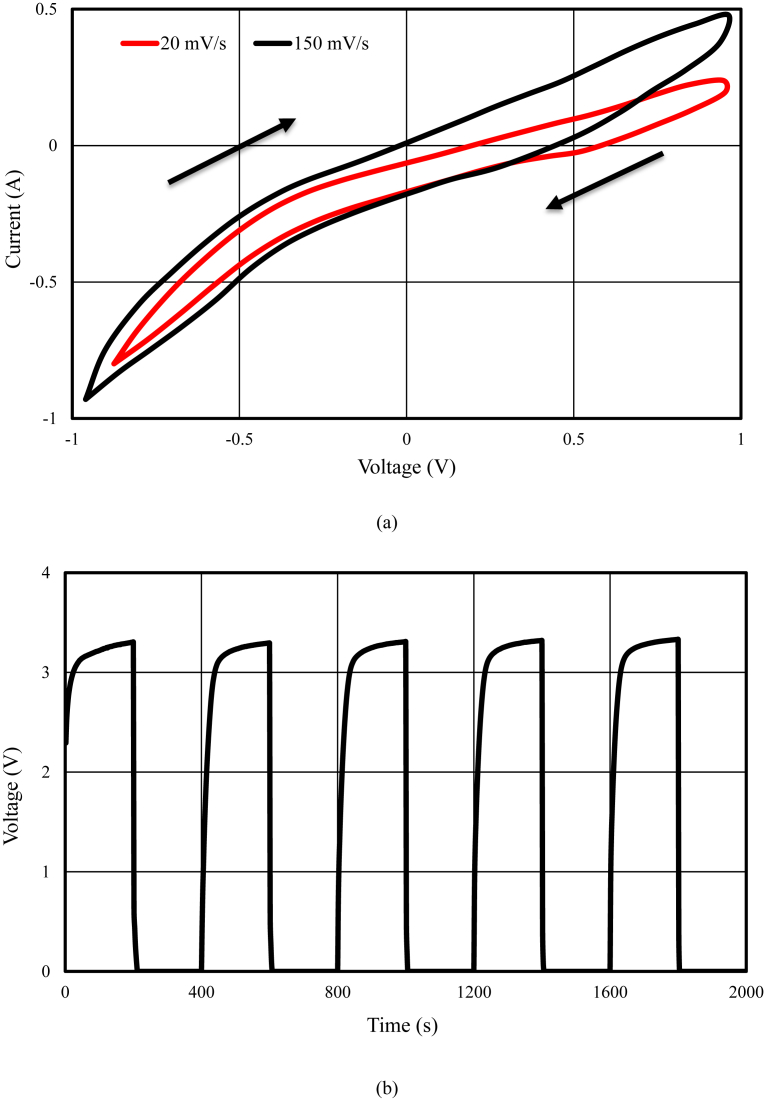
(a) Cyclic voltammetry (C-V) at 20 mV/s and 150 mV/s (b) Galvanostatic charge-discharge results from paper supercapacitor with the graphite nanoparticles electrodes.

The schematic of the symmetric paper supercapacitor (dimension 3 cm × 6 cm) with the structure of GNPs electrodes and also the image of the fabricated supercapacitor were illustrated in Fig. 1. As can be seen in Fig. 1, using the push coating, a thin layer of gel separator, electrolyte and electrode has been coated on both sides of the paper. The SEM images of the paper surface before and after the coating of BaTiO_3_ and also the PVA gel electrolyte surface and GNPs electrode surface morphology have been shown in Fig. 2. The SEM image of the BaTiO_3_ surface shows the pores of the surface were filled with gel separator. In the Fig. 2 (e), the TEM image of the GNPs has been shown. The average size of GNPs diameter analyzed by using Simagis Live [19] was 40 nm. The specific capacitance of the symmetric paper supercapacitor was measured using cyclic voltammetry and charge-discharge methods. The cyclic voltammetry technique has been done in voltage scan rates 20 mV s^−^^1^ and 150 mV s^−^^1^. The C-V curves of the paper supercapacitor in the potential range −1 V to +1 V were presented in Fig. 3(a). As it can be seen in Fig. 3(a), for scan rate 150 mV s^−^^1^ the current was increased from -1.1 A to 2.17 A and for the scan rate 20 mV s^−^^1^ the current was changed from -0.3 A to 0.62 A. The specific capacitance of the supercapacitors using cyclic voltammetry method can be obtained from the following formula [20]:

(1) $$C_{m}={\left[mS\left(V_{2}-V_{1}\right)\right]}^{-1}\int_{V1}^{V2}I\;dV$$

Where *S* is the voltage scan rate and *m* is the mass of the electrode materials. From Eq. (1) the specific capacitances of the symmetric paper supercapacitor with GNPs electrode for 150 mV s^−^^1^ and 20 mV s^−^^1^ were 79 F g^−^^1^ and 380 F g^−^^1^ respectively. Ping et al. [21] showed the specific capacitance of graphene supercapacitor is larger at low-speed voltage scan that due to slow charge/discharge. The charge-discharge curves for symmetric paper supercapacitor with current density 0.06 mA cm^−^^2^ were shown in Fig. 3(b). As it can be seen in Fig. 3(b) the behavior of the paper supercapacitor as like a conventional capacitor. The specific capacitance of the supercapacitor using galvanostatic method can be obtained the following formula [22, 23]:

(2) $$C_{m}=I{\left[m\left(\frac{dV}{dt}\right)\right]}^{-1}$$

**Fig. 4:**
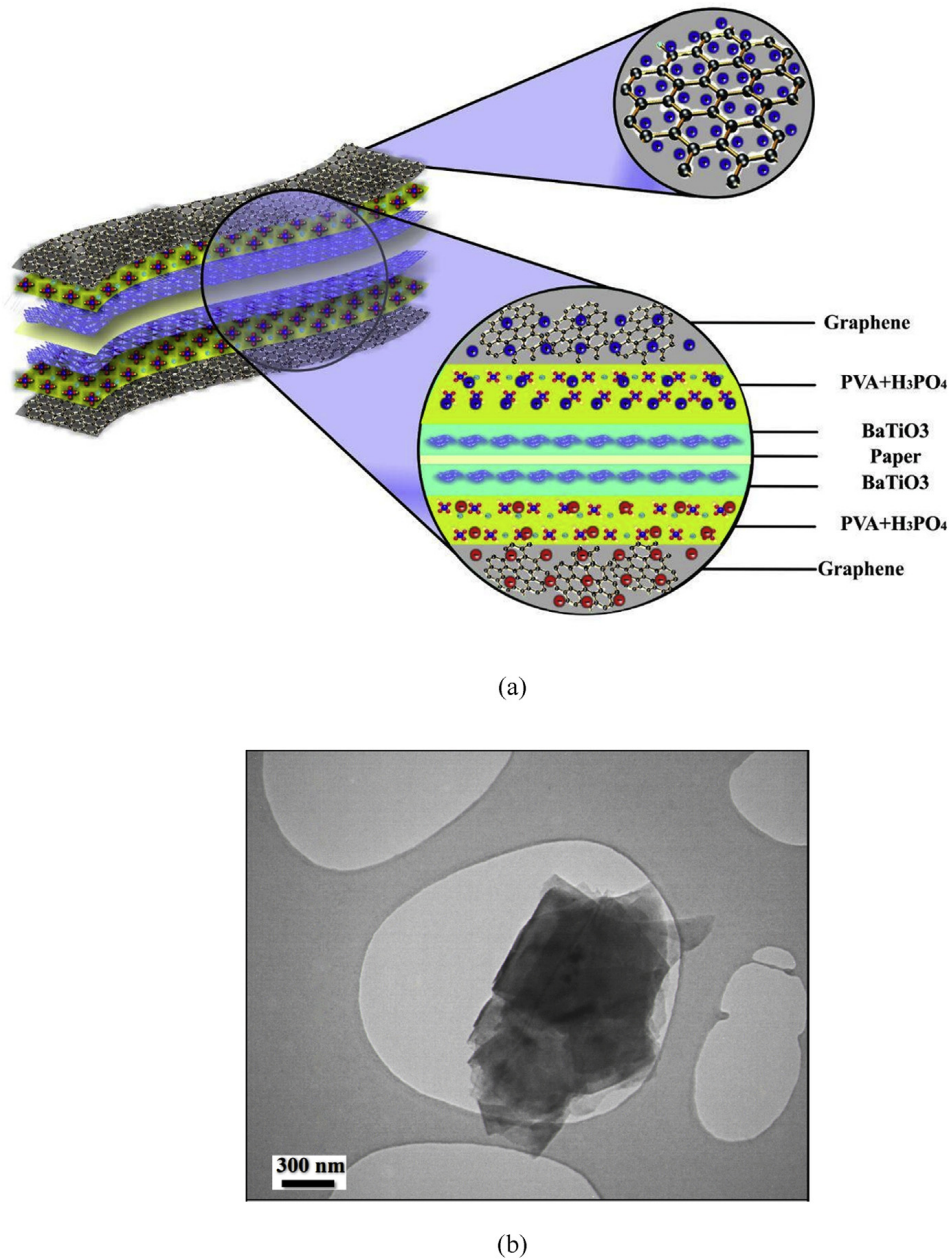
(a) Schematic of the paper supercapacitor with the graphene electrodes (b) TEM image of the graphene.

**Fig. 5:**
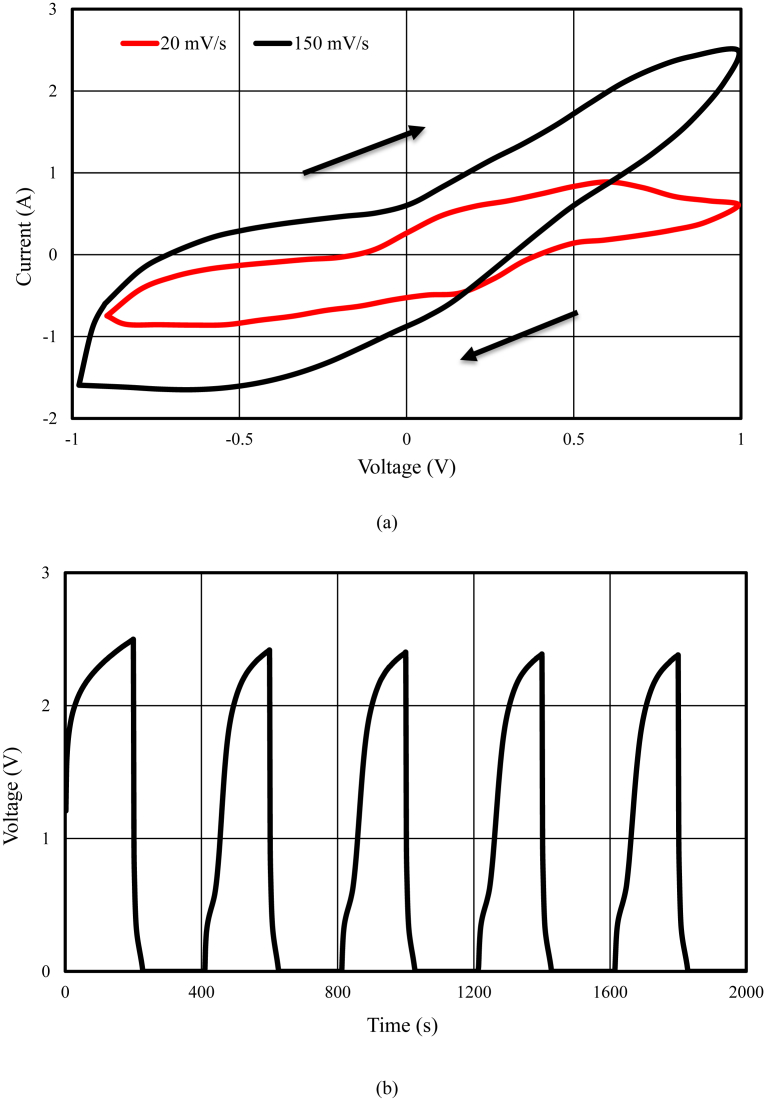
(a) Cyclic voltammetry (C-V) at 20 mV/s and 150 mV/s (b) Galvanostatic charge-discharge results from paper supercapacitor with the graphene electrodes.

**Fig. 6:**
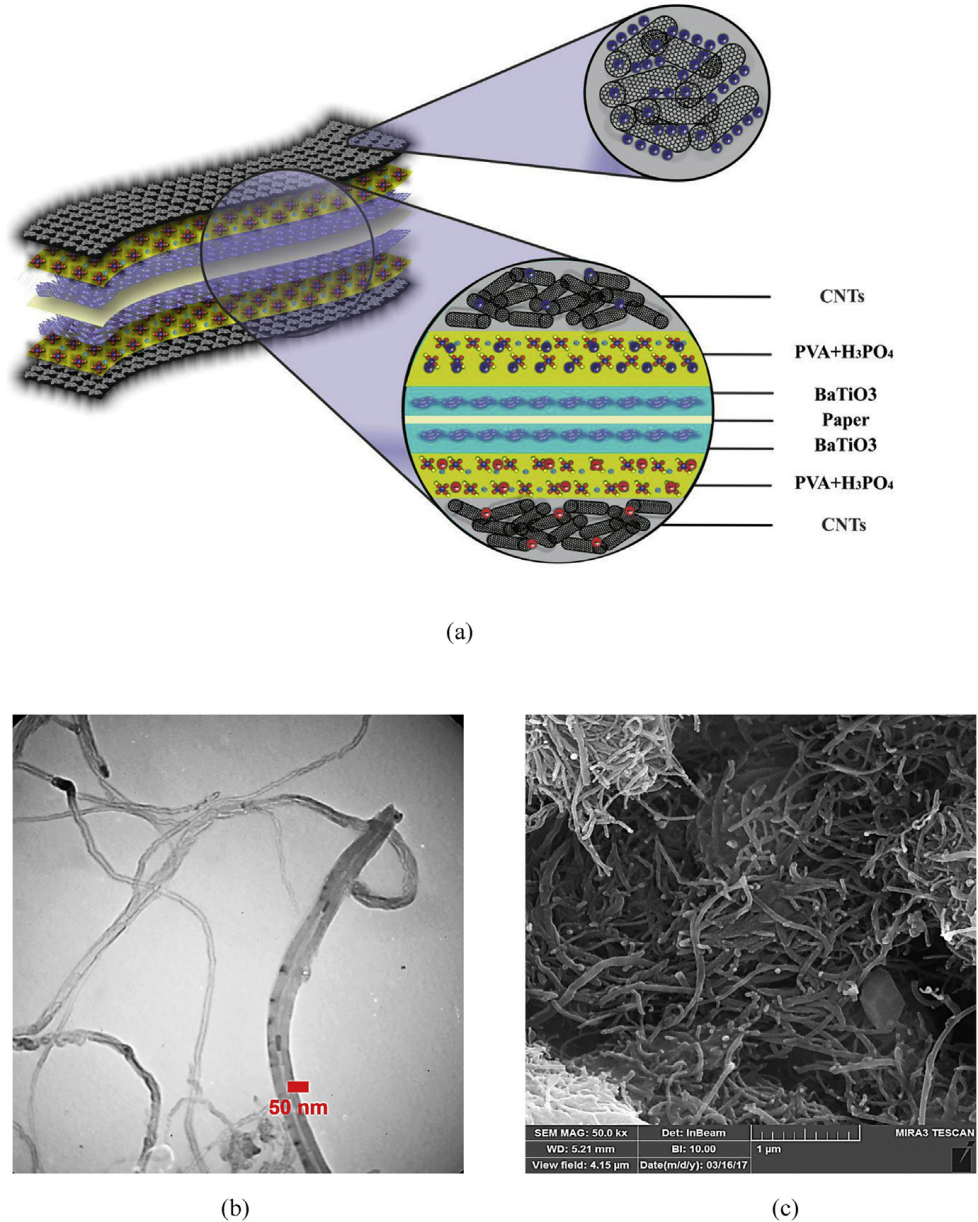
(a) Schematic of the paper supercapacitor with the CNTs electrodes (b) TEM image of the CNTs (c) SEM image of CNTs film surface.

**Fig. 7:**
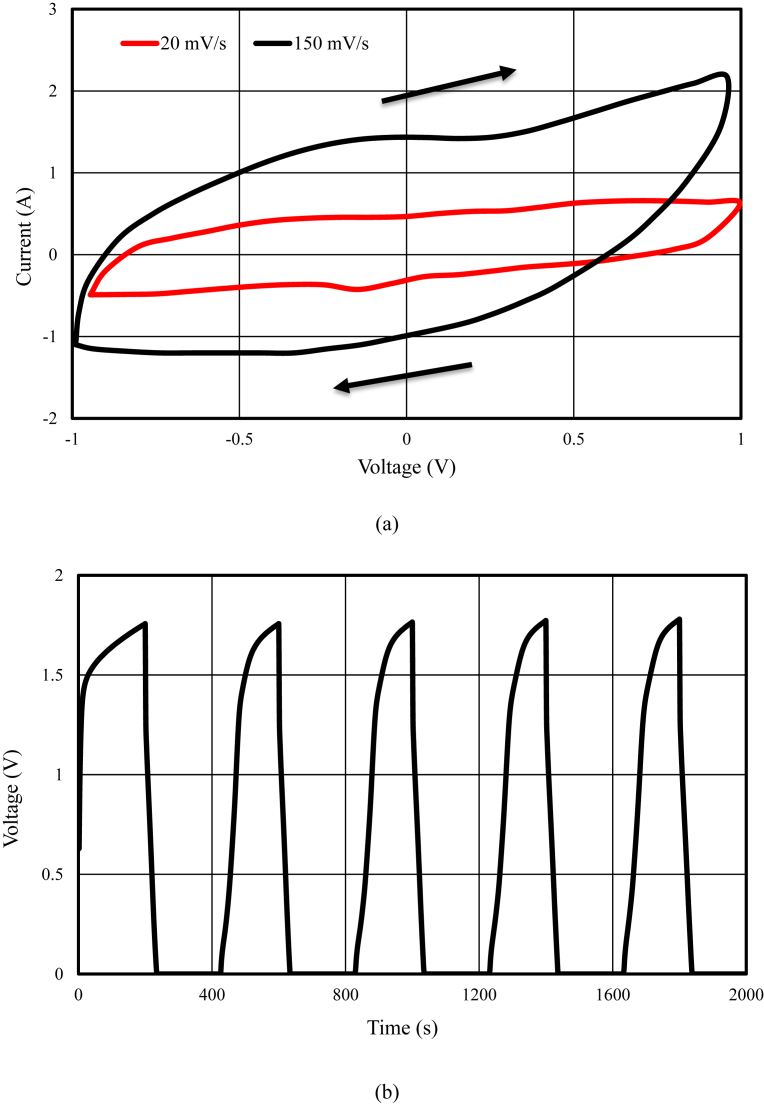
(a) Cyclic voltammetry (C-V) at 20 mV/s and 150 mV/s (b) Galvanostatic charge-discharge results from paper supercapacitor with CNTs electrodes.

Where *dv/dt* is the absolute value of the slope of the discharging curve. Using the Eq. (2) the specific capacitance of the paper supercapacitor was 163 F g^−^^1^ that is inside the range of the capacitance of C-V curves. The schematic of the symmetric paper supercapacitor based on the graphene electrodes is shown in Fig. 4 (a). In this structure using push coting method on the gel electrolyte film, a layer of the graphene has been coated. The TEM image of the graphene has been shown in Fig. 4 (b). The C-V curves of the symmetric paper supercapacitor based on the graphene electrode were illustrated in Fig. 5(a). From Eq. (1) the specific capacitances of the symmetric paper supercapacitor with graphene electrode for 150 mV s^−^^1^ and 20 mV s^−^^1^ were 87 F g^−^^1^ and 410 F g^−^^1^ respectively. The charge-discharge curves for paper supercapacitor based on graphene electrodes were presented in Fig. 5 (b). From Eq. (2) the specific capacitance of the paper supercapacitor was 310 F g^−^^1^ that is inside the range of the capacitance of C-V curves. Dai et al. [24], showed the specific capacitance for the paper supercapacitor based on the KCu_7_S_4_/graphene electrode and PVA electrolyte was 483 F g^−^^1^. The supercapacitor based on the graphene-PEDOT electrode has been investigated in Ref. [25]. That paper described the specific capacitance of the supercapacitor was estimated to be 374 F g^−^^1^. The schematic of the symmetric paper supercapacitor based on the CNTs electrodes was displayed in Fig. 6 (a). Fig. 6 (b) shows the TEM image of the CNTs. The CNTs diameter were analyzed using Simagis Live [19] and were around 7–55 nm. The average size of CNTs diameter was 15 nm. Using spry method, a thin layer of CNTs has been coated on the gel electrolyte film. The SEM images of CNTs electrode surface have been shown in Fig. 6 (c). The C-V curves of the symmetric paper supercapacitor based on CNTs electrodes were presented in Fig. 7(a). The C-V curve of the symmetric paper supercapacitor at 150 mV s^−^^1^ showed a nearly rectangular shape. From Eq. (1) the specific capacitances of the symmetric paper supercapacitor with CNTs electrode for 150 mV s^−^^1^ and 20 mV s^−^^1^ were 164 F g^−^^1^ and 411 F g^−^^1^ respectively. The charge-discharge curves for the symmetric paper supercapacitor based on CNTs electrodes were illustrated in Fig. 7 (b). From Eq. (2) the specific capacitance of the paper supercapacitor was 520 F g^−^^1^. Comparison of the specific capacitance of the symmetric paper supercapacitors based on graphite nanoparticles, graphene and CNTs electrodes shows that the capacitance of the CNTs supercapacitor is higher than the graphene and graphite nanoparticles supercapacitor. Kalam et al. [26], reported the paper supercapacitor based on CNTs electrode and the PVA gel at the voltage scan rate of 150 mV/s. They showed the specific capacitance of the paper supercapacitor was 219 F g^−^^1^. In another study, the polyester paper supercapacitor has been investigated based on the CNTs electrode and PVA gel electrolyte by Karthika et al. [27]. They reported the specific capacitance of the paper supercapacitor was 276 F g^−^^1^ at 5 mV/s. The energy density and the power density can be obtained from [28]:

(3) $$E\left(\frac{Wh}{Kg}\right)=\frac{1}{2}\frac{1000}{3600}C_{S}V^{2}$$

(4) $$P\left(\frac{W}{Kg}\right)=\frac{E\times 3600}{t}$$

**Fig. 8:**
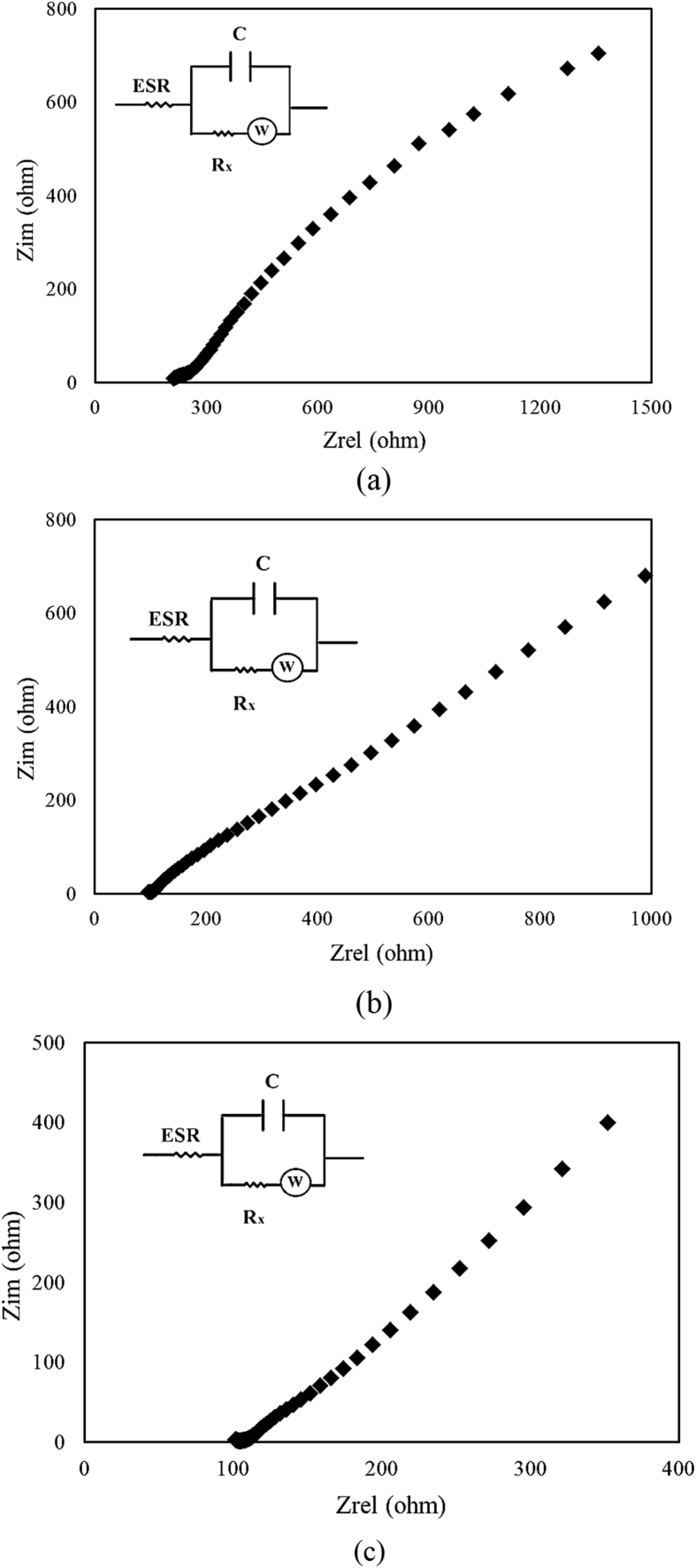
Nyquist curves of the symmetric paper supercapacitors based on the (a) graphite nanoparticles electrode (b) graphene electrode (c) CNTs electrode.

**Fig. 9:**
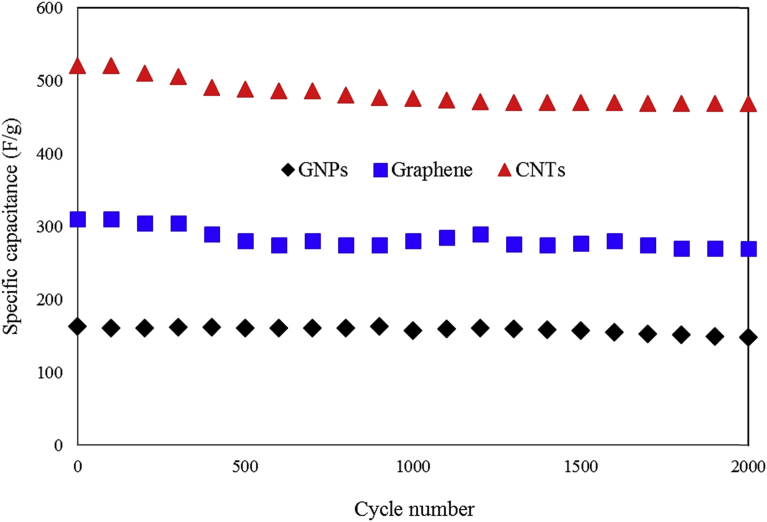
The specific capacitance of the paper supercapacitors as function of cycle number.

Where *V* is the potential window in the discharge process. The *C*_*S*_ is the specific capacitance of the paper supercapacitor and *t* is the discharge time. The energy density and power density of the GNPs-based supercapacitor were 79 Wh/Kg and 11 KW/Kg respectively. For graphene-based supercapacitor and CNTs-based supercapacitor the energy density were 81 Wh/Kg and 91 Wh/Kg and power density were 12 KW/Kg and 13.6 KW/Kg respectively. The equivalent circuit of the symmetric paper supercapacitor is the capacitor (C) in parallel with the resistance (R_x_). This resistance is due to self-discharge in the supercapacitors. The equivalent series resistance (ESR) is series with the capacitor. The ESR is related to the resistance of the electrodes. The equivalent circuit of the symmetric paper supercapacitor was plotted in Fig. 8. The resistance of the symmetric paper supercapacitors was measured using the electrochemical impedance spectroscopy. The electrochemical impedance spectroscopy was done at a dc bias of 10 mV with the frequency range of 100 KHz to ∼ 10 mHz. The Nyquist curves of the symmetric paper supercapacitors based on GNPs, graphene and CNTs electrodes were compared in Fig. 8(a,b,c). The equivalent series resistance of the supercapacitor can be obtained from the Nyquist curve. In the 100 kHz frequency, the real part of the impedance was denoted as ESR. For the symmetric paper supercapacitors based on the GNPs, graphene and CNTs electrodes the ESR resistance was 210 Ω, 96 Ω and 101 Ω respectively. The Nyquist curve slope is related to the Warburg resistance [29]. This resistance is due to the frequency dependency of the ion diffusion from the electrolyte to the electrode. Comparison of ESR resistance showed the resistance of graphene electrode is smaller than the CNTs and GNPs electrodes. Fig. 9 shows the specific capacitance of the paper supercapacitors as function of cycle number. The cycling stability for the paper supercapacitors with GNPs, graphene and CNTs electrodes were carried out by charge/discharge experiments for 2000 cycles. The paper supercapacitors based on the carbon nanomaterials exhibit good cyclic stability after 2000 charge/discharge cycles. This result indicates that the paper supercapacitors based on the PVA gel electrolyte is stable, and could be considered in the application of energy-storage devices.

## 4 Conclusion

In this study, the symmetric paper supercapacitors with the structures of CNTs, GNPs and graphene electrodes have been fabricated. The specific capacitance of the symmetric paper supercapacitor based on GNPs, graphene and CNTs electrodes for voltage scan rate (150 mV s-1 -20 mV s-1) were (79 F g-1 -380 F g-1), (87 F g-1-410 F g-1) and (164 F g-1-411 F g-1) respectively. The specific capacitance of the symmetric paper supercapacitor based on GNPs, graphene and CNTs electrodes, using the charge-discharge method were 163 F g-1, 310 F g-1, and 520 F g-1 respectively. The energy density and power density of the GNPs-based supercapacitor were 79 Wh/Kg and 11 KW/Kg respectively. For graphene-based supercapacitor and CNTs-based supercapacitor the energy density were 81 Wh/Kg and 91 Wh/Kg and power density were 12 KW/Kg and 13.6 KW/Kg respectively. The resistance of the symmetric paper supercapacitors was measured using the electrochemical impedance spectroscopy. The results of the symmetric paper supercapacitors based on the nanomaterials electrode showed the CNTs have an excellent performance that could be applied for electrical energy storage.

## Declarations

### Author contribution statement

L. Fekri Aval: Conceived and designed the experiments; Analyzed and interpreted the data; Wrote the paper.

M. Ghorannevis: Contributed reagents, materials, analysis tools or data.

G. Behzadi Pour: Performed the experiments; Analyzed and interpreted the data.

### Funding statement

This work was supported by the Iran National Science Foundation (INSF) and grant number (INSF: 96007938).

### Competing interest statement

The authors declare no conflict of interest.

### Additional information

No additional information is available for this paper.
